# Supplementary material for: WordCommentsAnalyzer: A windows software tool for qualitative research
Source: F1000Res. 2018 Sep 4;7:536. Originally published 2018 May 3. [Version 2] doi: 10.12688/f1000research.14819.2 (PMC6137411; doi:10.12688/f1000research.14819.2)
Supplement: Supplementary file 3 [file f1000research-7-17614-s0002.tgz › b2a79496-4ada-47c4-9682-a99ce867836d.zip!/Eight Iranian Health Journals/Salmand_Iranian Journal of Ageing _31.docx]

© Salmand_Iranian Journal of Ageing . This work is licensed under CC BY-NC 4.0 (https://creativecommons.org/licenses/by-nc/4.0/).

Volume & Issue: دوره 5 - شماره 4

Title: تأثیر مداخله آموزشی بر اساس مدل پرسید بر ارتقای کیفیت زندگی سالمندان

http://salmandj.uswr.ac.ir/article-1-264-fa.html

اهداف: هدف از انجام پژوهش، بررسی تاثیر مداخله آموزشی بر مبنای الگوی "پرسید" در ارتقاء کیفیت زندگی سالمندان بود. مواد و روش‌ها: پژوهش نیمه تجربی حاضر بر 54 نفر از سالمندان تحت پوشش فرهنگسرای سالمندان تهران (1388) انجام شد. سالمندان به روش نمونه گیری مستمر، در دو گروه مداخله و کنترل قرار گرفتند. برای مداخله آموزشی از پرسشنامه محقق ساخته . طراحی شده بر اساس سازه‌های الگوی اموزشی "پرسید" و برای سنجش کیفیت زندگی، از پرسشنامه کیفیت زندگی سازمان جهانی بهداشت استفاده شد. کیفیت زندگی سالمندان قبل و یک ماه بعد از مداخله آموزشی(چهار جلسه آموزش به سالمندان و یک جلسه آموزش برای کارکنان در خصوص شیوه‌های زندگی سالم) بررسی شد. داده‌ها با آزمون‌های آماری مک نمار، تی مستقل و تی زوجی بررسی شدند. یافته‌ها:‌ میانگین نمره کل اجزاء مدل "مدل پرسید" شامل عوامل مستعد کننده (آگاهی و نگرش)، عوامل قادر کننده، عوامل تقویت کننده، عوامل رفتاری و در نتیجه کیفیت زندگی سالمندان گروه مداخله در مقایسه با گروه کنترل، پس از مداخله آموزشی (P<0/05)  اختلاف معنی داری را نشان داد. نتیجه‌گیری: به نظر می رسد می توان با مداخله آموزشی الگوی "پرسید" کیفیت زندگی سالمندان را افزایش داد.

Title: تأثیر 12 هفته تمرین عملکردی بر تعادل پویای زنان سالمند سالم

http://salmandj.uswr.ac.ir/article-1-263-fa.html

اهداف: هدف پژوهش حاضر، بررسی تأثیر تمرینات عملکردی بر تعادل پویای زنان سالمند سالم بود. مواد و روش‌ها: در پژوهش نیمه تجربی حاضر 30 زن سالمند سالم به صورت هدفمند انتخاب و سپس به طور تصادفی به دو گروه مساوی کنترل و تجربی تقسیم شدند. تعادل پویای آزمودنی­‌ها با تست عملکردی «زمان برخاستن و رفتن» اندازه‌گیری شد.  گروه تجربی به مدت سه جلسه در هفته در یک برنامه 12 هفته‌ای تمرینات عملکردی شرکت کردند. در حالی که گروه کنترل در هیچ گونه تمرین ورزشی شرکت نداشتند. از آزمون T  همبسته برای بررسی تفاوت درون‌گروهی و از آزمون T مستقل برای بررسی تفاوت بین‌گروهی در سطح معناداری (P<0/05) استفاده شد. یافته‌ها: تعادل پویای گروه کنترل بعد از 12 هفته تمرین عملکردی تغییر معنی‌داری نداشت (P=0/11). زمان آزمون «برخاستن و رفتن» به عنوان شاخص تعادل پویا، در گروه تجربی از لحاظ درون‌گروهی و بین‌گروهی به طور معنی‌داری کاهش یافت (P=0). نتیجه‌گیری: تمرینات عملکردی می تواند تعادل پویای زنان سالمند را به نحو مؤثری بهبود بخشیده نقش به سزایی در زندگی سالم زنان سالمند داشته باشد.

Title: مقایسه کیفیت زندگی و سلامت روان در سالمندان ساکن/غیر ساکن سرای سالمندان

http://salmandj.uswr.ac.ir/article-1-266-fa.html

اهداف: پژوهش حاضر باهدف مقایسه سلامت روان و کیفیت زندگی سالمندان ساکن سرای سالمندان و سالمندان عضو کانون بازنشستگی صورت گرفت. مواد و روش‌ها: در پژوهش علی مقایسه ای حاضر، جامعه آماری شامل کلیه سالمندان ساکن سرای سالمندان و سالمندان عضو کانون بازنشستگی شهر شیراز (1389) بود. نمونه آماری شامل 60 سالمند (30 مرد و 30 زن) ساکن دو سرای سالمندان که از طریق نمونه گیری خوشه ای تصادفی انتخاب و60  سالمند (30 مرد و 30 زن)  عضو کانون بازنشستگی بود که از طریق گروه های جور شده انتخاب شدند. از پرسشنامه های سلا مت عمومی (GHQ-28)، پرسشنامه کیفیت زندگی (SF-36)، و مشخصات جمعیت شناختی استفاده شد. داده‌ها با ازمون t مستقل و تحلیل واریانس دوطرفه بررسی شدند. یافته‌ها: نتایج پژوهش نشان داد که بین دو گروه سالمند در متغیر های کیفیت زندگی (t=8/41 و P<0/001 )  و سلامت روان (t=16/4 و P<0/001) و تمام اندازه های خرده مقیاس این دو متغیر تفاوت معنی‌داری وجود دارد. همچنین اثرات معنی دار مستقلی برای متغیر های سن، جنس، شغل و سطح اقتصادی در تعیین واریانس دو متغیر کیفیت زندگی و سلامت روان مشاهده شد. نتیجه‌گیری: یافته های پژوهش حاکی از تفاوت معنی دار فاحش بین کیفیت زندگی، سلامت روان و کلیه خرده مقیاس های این دو متغیر بود . و در همه موارد، برتری با سالمندان عضو کانون بازنشستگی بود . علاوه بر این شواهد، حاکی از آن است که متغیر های جمعیت شناختی می تواند بر اثر محل سکونت نقش تعدیل کننده د اشته باشند. هرچند میزان این تعدیل کنندگی چندان قابل ملاحظه نیست.

Title: تأثیر تمرینات منتخب هاتا یوگا بر تعادل زنان سالمند

http://salmandj.uswr.ac.ir/article-1-265-fa.html

اهداف: این مطالعه با هدف تعیین اثربخشی تمرین های هاتایوگا بر تعادل ایستا و پویای زنان سالمند صورت گرفت. مواد و روش‌ها: جامعه آماری این مطالعه نیمه تجربی را زنان سالمند شهر مشهد (1388) تشکیل دادند که داوطلب شرکت در پژوهش بودند. 29 زن سالمند سالم (دامنه سنی 56-72 سال) به روش در دسترس و داشتن ملاک‌های ورود (عدم وجود سابقه بیماری پوکی استخوان پیشرفته، جایگزینی لگن، کوری تدریجی، پارکینسون، سرگیجه، و جراحی در یک سال گذشته) در نمونه آماری قرار گرفتند.  تعادل ایستا و پویا به ترتیب از طریق آزمون ثبات وضعیتی و خطر افتادن به وسیله دستگاه تعادلی بایودکس قبل و بعد از مداخله سنجیده شد .برنامه تمرینی هاتایوگا در سه جلسه (یک ساعت) درهفته و به مدت هشت هفته اجرا شد؛ تحلیل آماری داده ها با استفاده از آزمون‌های تحلیل واریانس و  t همبسته در سطح معناداری α=0.05 انجام شد. یافته‌ها: یافته های پژوهش نشان داد که برنامه تمرینی هاتا یوگا به مدت 24 جلسه تأثیر معناداری بر تعادل ایستا: ثبات کلی (P=0/001)، تعادل قدامی-خلفی (P=0/002)، تعادل داخلی-خارجی (P=0/006) و همچنین تعادل پویای زنان سالمند داشت (P=0/01). نتیجه‌گیری: با توجه به اثر بخشی برنامه تمرینی هاتایوگا بر تعادل ایستا و پویا به نظر می رسد برنامه تمرینی با بهبود قدرت عضلانی زنان سالمند به ویژه استقامت و قدرت عضلات در اندام تحتانی واحتمالاً با افزایش کارایی سیستم حرکتی و عصبی و تقویت سازوکار گیرنده های عمقی عضلات و مفاصل بر تعادل اثر داشته و به کاهش خطر زمین خوردن در زنان سالمند منجر شده است.

Title: مقایسه «اختصاص توجه» در راه‌رفتن و انجام تکلیف ثانویه شناختی‌حرکتی در سالمندان با / بدون سابقه زمین‌خوردن

http://salmandj.uswr.ac.ir/article-1-260-fa.html

اهداف: هدف اصلی این مطالعه مقایسه توانایی اختصاص «توجه» بر تکلیف اولیه و ثانویه شناختی یا حرکتی در سالمندان با و بدون سابقه زمین خوردن بود. مواد و روش‌ها: مطالعه علّی مقایسه‌ای حاضر با طرح سه عاملی مختلط بر نمونه‌ای متشکل از 55 نفر اجرا شد. شرکت‌کنندگان به روش نمونه‌گیری غیراحتمالی ساده انتخاب شدند. 23 سالمند با میانگین سنی 72/3 سال و گزارش حداقل یک مرتبه زمین خوردن غیرترقبه در طی سال گذشته و 33 سالمند بدون سابقه زمین خوردن با  میانگین سنی 69/12 سال شرکت کردند. مطالعه بر سالمندان ساکن آسایشگاه‌های تهران انجام شد. از آزمون‌های بررسی وضعیت روانشناختی  (MMSE) و زمان نشستن و برخاستن (TUG) همراه با تکالیف ثانویه شناختی و حرکتی استفاده شد. شرایط آزمون به صورتی بود که شرکت‌کنندگان ابتدا می‌بایست ضمن انجام تکلیف دوگانه به تکلیف اولیه توجه داشته باشند و سپس می‌بایست در ضمن انجام تکلیف دوگانه توجه خود را معطوف تکلیف ثانویه می کردند. زمان و تعداد قدم‌ها در آزمون TUG با دوربین فیلمبرداری ثبت شد. داده‌ها با آزمون تحلیل واریانس مختلط برای سنجش های مکرر 2×2×2 (گروه با دو سطح، توانایی اختصاص توجه با دو سطح، و تکلیف ثانویه با دو سطح بر متغیر وابسته زمان آزمون TUG) بررسی شدند. یافته‌ها: نتایج اثر خالص معنی‌دار گروه، توانایی اختصاص توجه، تکلیف ثانویه بر زمان و تعداد قدم‌ها در آزمون TUG (P<0/001) را نشان داد. اثر متقابل گروه با نوع تکلیف ثانویه بر زمان وتعداد قدم‌ها در آزمون TUG معنی‌دار (P=0/74  و P=0/39) نبود. اثر متقابل گروه با اختصاص توجه بر زمان و تعداد قدم‌ها در آزمون  TUG معنی‌دار (P<0/001) بود. اثر متقابل نوع تکلیف ثانویه با اختصاص توجه بر زمان در آزمون TUG معنی دار (P=0/001) بود، ولی بر تعداد قدم‌ها معنی‌دار (P=0/4) نبود. نتیجه‌گیری: در تحقیق حاضر سالمندان با و بدون سابقه زمین خوردن توانایی اختصاص توجه به تکلیف اولیه را داشتند که شاید بتوان ادعا کرد سالمندان به حفظ تعادل و راه رفتن بیشتر از انجام تکلیف ثانویه اهمیت می‌دهند .گروه سالمندان بدون سابقه زمین خوردن توانایی اختصاص توجه بهتری نسبت به گروه سالمندان با سابقه زمین خوردن نشان دادند. نداشتن توانایی اختصاص توجه یکی از عوامل مهم در اختلال تعادل و زمین خوردن است.

Title: رابطه جهت‌گیری مذهبی و شادکامی سالمندان

http://salmandj.uswr.ac.ir/article-1-267-fa.html

اهداف:  مطالعه حاضر با هدف بررسی رابطه جه تگیری مذهبی و شادکامی سالمندان انجام شد. مواد و روش‌ها: مطالعه علّی  مقایسه ای حاضر بر نمونه ای متشکل از 389 سالمند بالای 60 سال ( 247 مرد و142  زن) که به صورت خوشه ای چندمرحله ای از جامعه سالمندان شهرستان تهران انتخاب شده بودند، انجام شد. از پرسشنامه جهت گیری مذهبی آلپورت و سیاهه شادکامی آکسفورد استفاده شد. داده ها با آزمون همبستگی پیرسون و آزمون  Tبررسی شدند. یافته‌ها: نتایج نشان داد که در دو جنس بین جهت گیری مذهبی درونی و شادکامی رابطه مثبت و معنی‌داری (P<0/01) وجود دارد. میزان شادکامی در سالمندان با جهت‌گیری مذهبی درونی بیشتر از سالمندان با جهت‌گیری مذهبی بیرونی بود (P<0/01). رابطه بینجهت گیری مذهبی بیرونی و شادکامی در سالمندان معنی دار نبود (P<0/01). نمرات زنان سالمند در هر دو جهت گیری مذهبی درونی و بیرونی بیشتر از مردان سالمند بود. شادکامی زنان سالمند بیشتر از مردان سالمند بود (P<0/01). نتیجه‌گیری: نتایج پژوهش حاضر نشان داد رابطه جهت گیری مذهبی درونی در سالمندان با شادکامی مثبت بود .در تائید اکثر مطالعات حوزه روان شناسی دین همچنین مشخص شد زنان سالمند در تحقیق حاضر از مرادن دین‌دارترند. تحقیقات بیشتر در حیطه متغیرهای مورد بررسی می توانند روشنگر باشند.

Title: اثربخشی پیاده‌روی بر عملکرد روانی‌اجتماعی مرتبط با کیفیت زندگی مردان سالمند

http://salmandj.uswr.ac.ir/article-1-261-fa.html

اهداف: هدف از پژوهش حاضر، بررسی تأثیر پیاده‌روی بر عملکرد روانی اجتماعی مردان سالمند بود. مواد و روش‌ها: پژوهش حاضر از نوع شبه‌تجربی با طرح پیش‌آزمون و پس‌آزمون با گروه کنترل بود. جامعه آماری پژوهش را سالمندان مرد 60 تا  75 سال ساکن سرای سالمندان کهریزک تشکیل دادند که از نظر جسمانی و سابقه پزشکی سالم ارز یابی شدند. تعداد 80 نفر از سالمندان با توجه به ملاک‌های ورود، انتخاب و به طور  تصادفی به سه گروه تجربی (1، 3 و 6 جلسه در هفته، ، هر جلسه 30 دقیقه پیاده‌روی با شدت متوسط) و یک گروه کنترل (بدون فعالیت بدنی در طی 16 هفته) تقسیم شدند. دو پرسشنامه فرم کوتاه کیفیت زندگی مرتبط با سلامتی (SF-36) و پرسشنامه سنجش علائم افسردگی (GDS-SF) در دو مرحله پیش‌آزمون و پس‌آزمون توسط شرکت‌کنندگان تکمیل شد. ملاک‌های ورود آزمودنی‌ها عبارت بودند از: دامنه سنی، داشتن رضایت و تمایل به حضور فعال و منظم در برنامه پیاده‌روی و برخورداری از سلامت روانی نسبی؛ وهمچنین نداشتن بیماری‌های دیابت، قلبی عروقی، پارکینسون، ناهنجاری‌های وضعیتی حاد، اختلالات عصب شناختی، اسکلتی عضلانی، فقدان سابقه بیماری‌های جدی به‌ویژه نقص بینایی، نقص در سیستم‌های درگیر در تعادل، نداشتن محدودیت حرکتی، ناهنجاری‌های کف پا، سابقه جراحی. داده‌ها با آزمون‌های تحلیل واریانس یک طرفه، همبستگی پیرسون و تعقیبی شفه در سطح معنی‌داری P<0/05 بررسی شدند. یافته‌ها: یافته‌ها نشان دادند عملکرد روانی اجتماعی مردان 60 تا 75 سال، پس از 16 هفته پیاده‌روی افزایش داشت (F=29/400 و P=0/001). و با افزایش جلسات هفتگی تمرین، اثربخشی پیاده‌روی نیز بیشتر شد. در حالی که بین گروه‌های مداخله با تمرین 1 جلسه و 3 جلسه در ارتباط با بهبود عملکرد روانی اجتماعی تفاوت معنی‌داری مشاهده نشد (P>0/05). نتیجه‌گیری: انجام پیاده‌روی منظم با مقدار تمرینی اثربخش و استاندارد برای سالمندان می‌تواند زمینه ارتقای کیفیت زندگی آنان را فراهم سازد. از این رو طرح‌ریزی فعالیت‌های بدنی نظام‌دار و منطقی برای سالمندان زیر نظر مربیان آگاه بر پایه یافته‌های تحقیق پیشنهاد می‌شود.

Title: اختلالات انسدادی دفعی و عوامل موثر در زنان بالای 40 سال: مطالعه همه‌گیرشناختی

http://salmandj.uswr.ac.ir/article-1-259-fa.html

اهداف: هدف این مطالعه اپیدمیولوژیک، تعیین میزان شیوع اختلالات انسدادی دفعی و عوامل مؤثر بر آن در زنان 40 سال به بالا بود. مواد و روش‌ها: در این مطالعه، مقطعی 800 زن 40 سال و بالاتر ساکن منطقه 17 تهران به روش نمونه‌گیری خوشه‌ای انتخاب و با مصاحبه و تکمیل پرسشنامه (منزل به منزل) بررسی شدند. برای آنالیز داده‌ها از آزمون خی دو و آزمون تعقیبی فیشر استفاده شد. یافته ها: در این مطالعه شیوع اختلال انسدادی دفع مدفوع خفیف 36/5% (حدود اطمینان 95%، 39%- 33)، متوسط 8% (حدود اطمینان 95%، 10%- 6%) و شدید 5/0% ( حدود اطمینان 95%، 9/0%- 01/0%) بود. اختلالات انسدادی دفع مدفوع فقط با سن رابطه معنی‌دار آماری داشت (P=0/006). با افزایش میزان تحرک بدنی نسبت موارد اختلال دفع کاهش نسبی نشان داد و این روند کاهشی از نظر آماری معنی‌دار بود (P=0/004). بین اختلال انسدادی دفع مدفوع و بیماری‌های آرتروز، افسردگی و اضطراب، پوکی استخوان، سرفه مزمن، بیماری روده‌ای مزمن، و دیابت رابطه معنی‎داری وجود داشت (P<0/005) نتیجه گیری: این مطالعه با مشخص کردن میزان شیوع قابل توجه اختلالات انسدادی دفعی و عوامل مؤثر بر آن در زنان جامعه ایرانی نشان داد که در باره این همه‌گیری پنهان و عوامل خطر و بازدارنده آن در بین زنان جامعه اطلاعات کمی وجود دارد و به ارزیابی و تحقیق بیشتری نیاز است. در مطالعه حاضر توجه به امر پیشگیری، غربالگری، آموزش و درمان به موقع بیماران برای ارتقای سلامت و کیفیت زندگی زنان توصیه می‌شود.
